# Supplementary material for: Bri2 BRICHOS client specificity and chaperone activity are governed by assembly state
Source: Nat Commun. 2017 Dec 12;8:2081. doi: 10.1038/s41467-017-02056-4 (PMC5727130; doi:10.1038/s41467-017-02056-4)
Supplement: Supplementary file 1 — Supplementary Information [file 41467_2017_2056_MOESM1_ESM.pdf]

Supplementary Figures

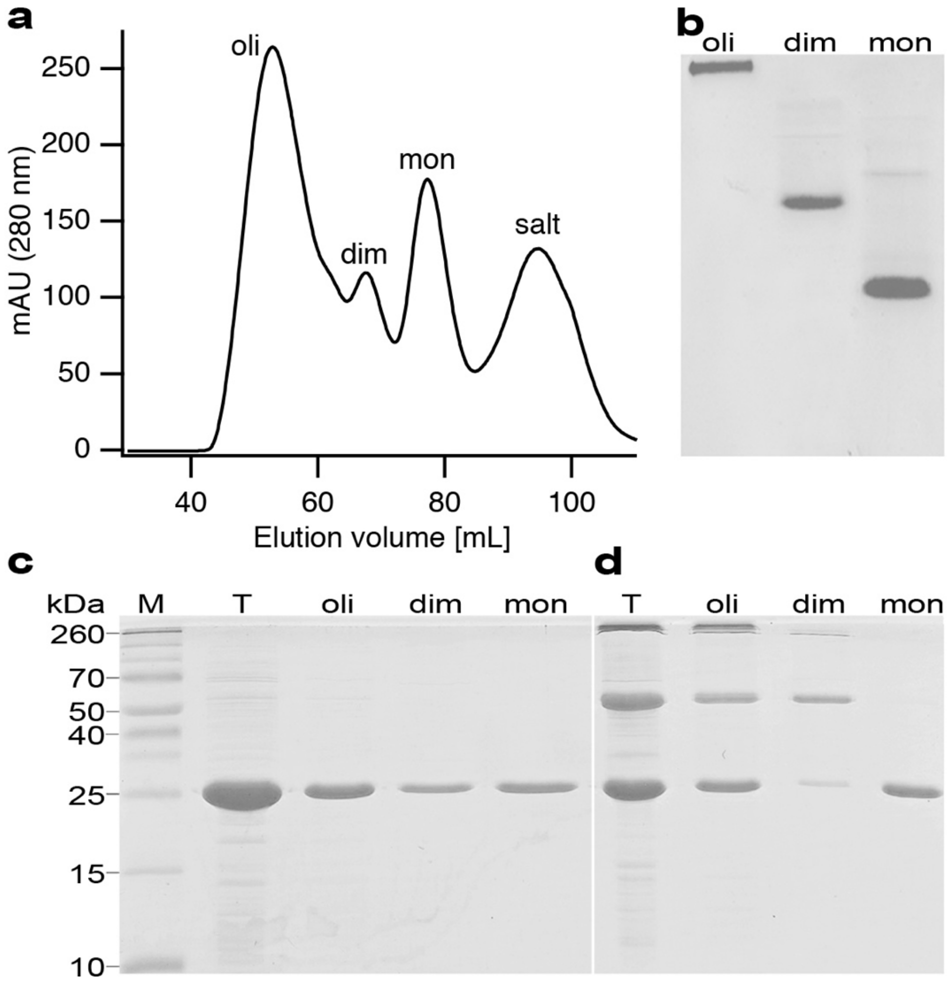

Supplementary Figure 1. SEC of recombinant NT*-Bri2 BRICHOS and PAGE analysis of the fractions. (a) NT*-Bri2 BRICHOS was separated on a Superdex 200 PG column, and oligomers (oli), dimers (dim) and monomers (mon) were collected and analyzed with native PAGE **(b)**, SDS-PAGE under reducing conditions **(c)**, and non-reducing conditions **(d)**. Lane M shows migration of protein size markers with masses indicated to the left. T stands for unresolved NT*-Bri2 BRICHOS proteins before SEC.

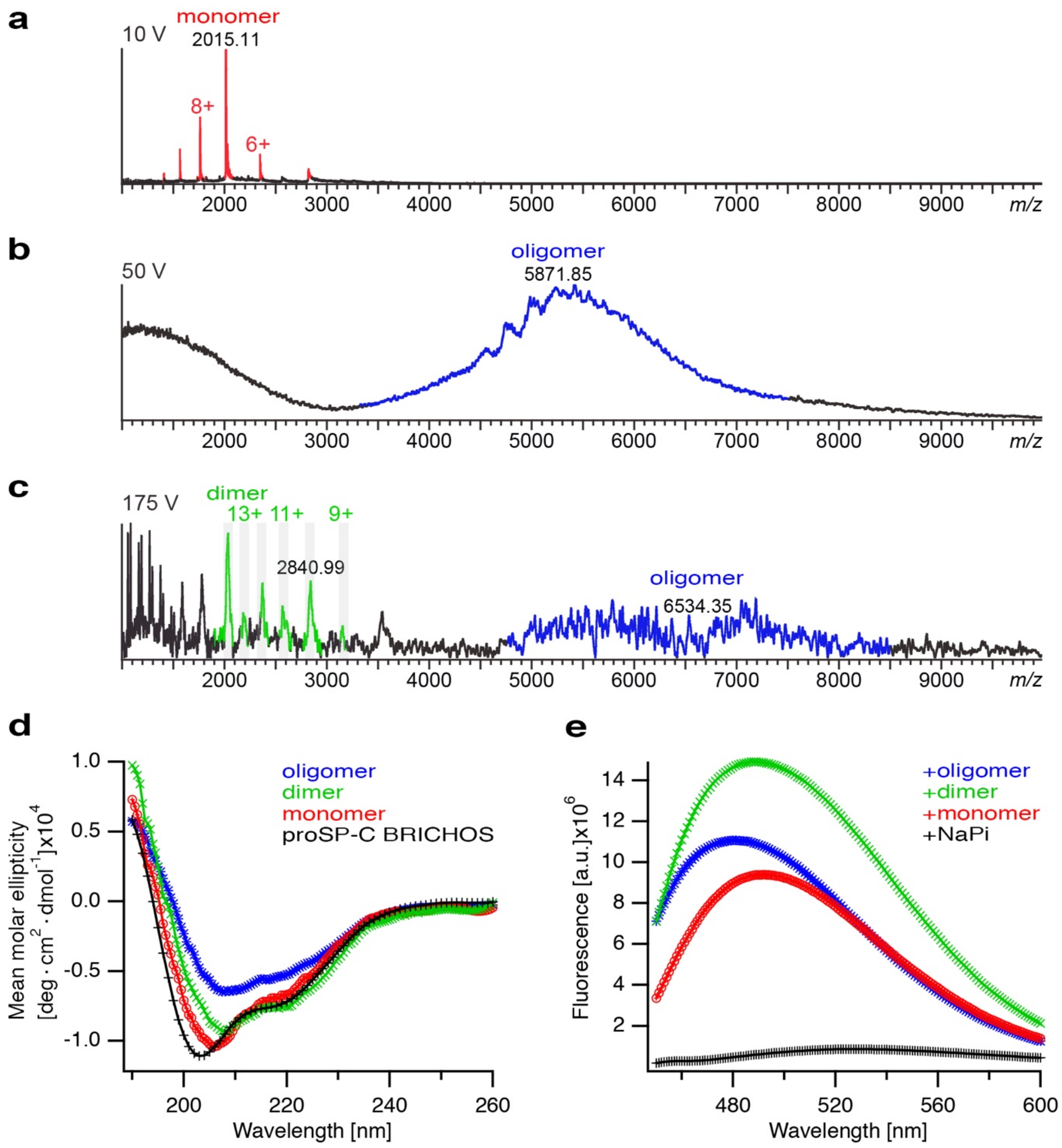

Supplementary Figure 2. Characterization of rh Bri2 BRICHOS species. Electrospray mass spectra of rh Bri2 BRICHOS monomers **(a)**, and oligomers at 50 V **(b)** or 175 V **(c)** collision voltage. **(d)** CD spectra at 25°C of rh Bri2 BRICHOS oligomer (blue), dimer (green), monomer (red), and proSP-C BRICHOS (black). **(e)** Fluorescence emission of 10 μM bis-ASN in sodium phosphate buffer (black) or in the presence of 2 μM rh Bri2 BRICHOS oligomer (blue), dimer (green), or monomer (red).

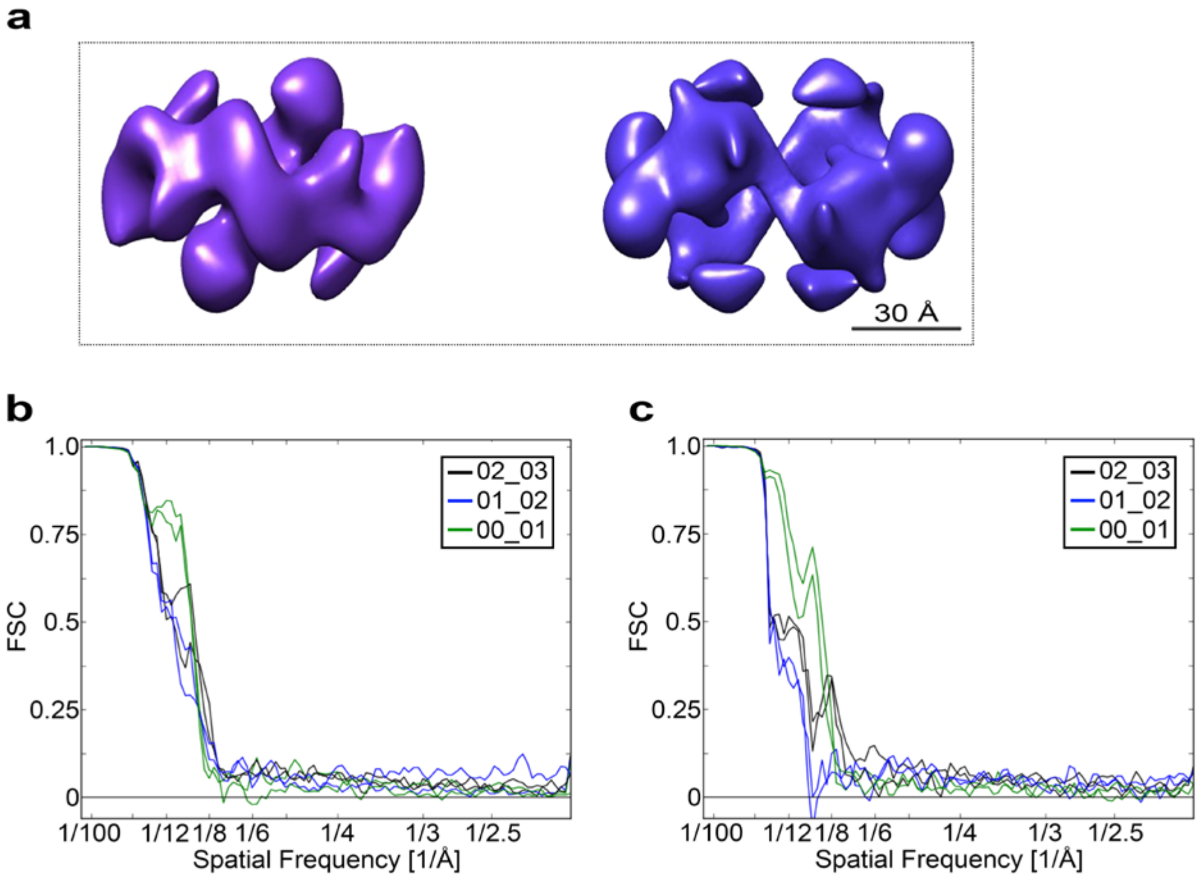

Supplementary Figure 3. Electron micrograph and single particle analysis of Bri2 oligomers. (a) Refinement with full sized data (1.038 Å/pixel) shows significant similarities between the 3D density maps with C2 symmetry (left) and D2 symmetry (rigth). Convergence plots for the maps with **(b)** C2 and **(c)** D2 symmetries (1.038 Å/pixel).

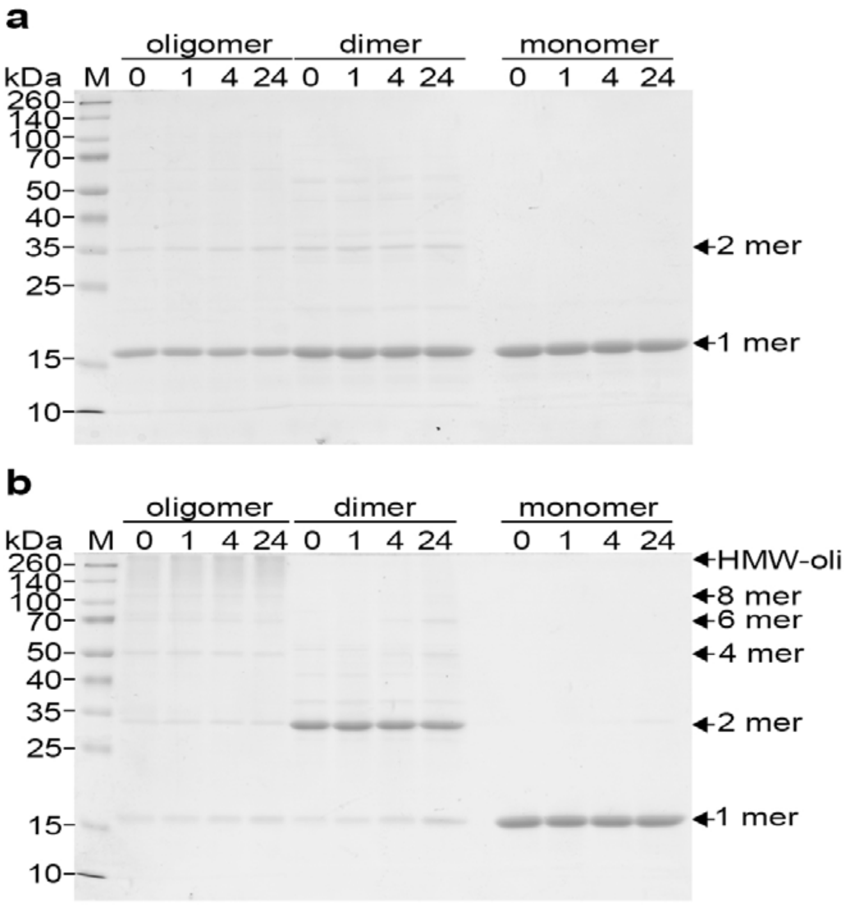

Supplementary Figure 4. Different assembly states of rh Bri2 BRICHOS species after incubation. The Bri2 BRICHOS oligomers, dimers and monomers at concentrations that are 2-3 times higher than the ones used in the ThT assay are incubated in buffer as the same as the ThT assay at 37℃. Samples were analyzed for assembly state by SDS-PAGE after 0, 1, 4 and 24 hours under reducing (**a**) and non-reducing conditions (**b**).

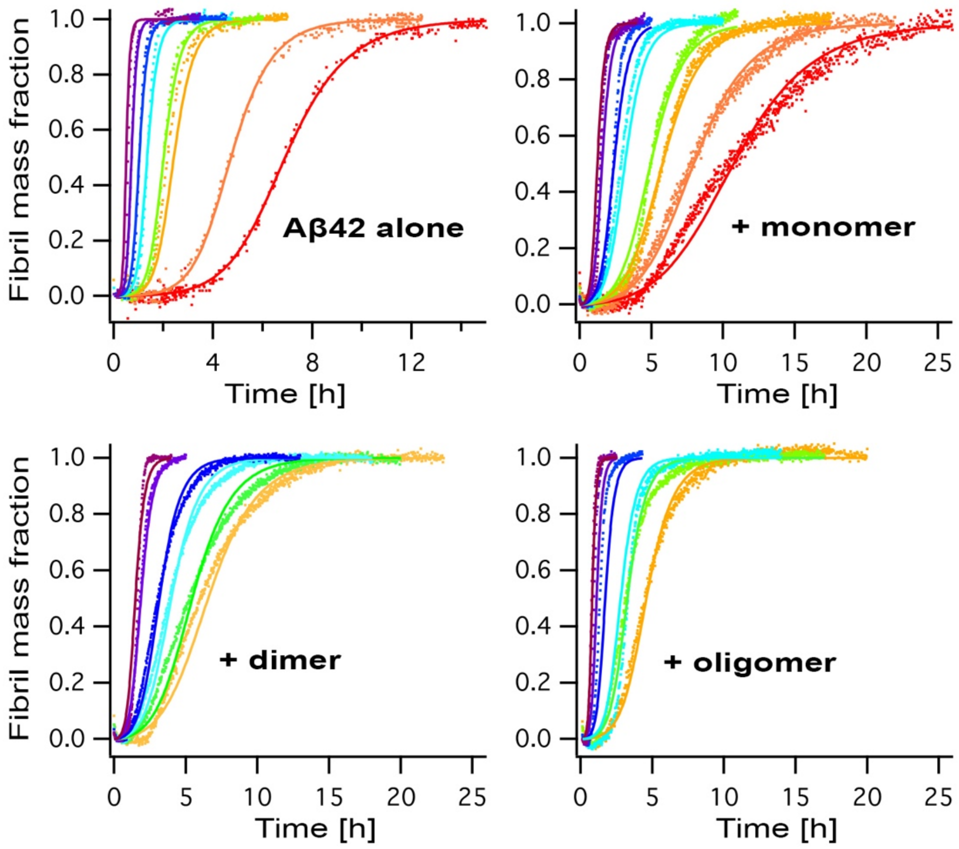

Supplementary Figure 5. Global fits of aggregation traces of Aβ42 in the presence and absence of rh Bri2 BRICHOS species. Global fits (solid lines) of aggregation traces (dots) at different peptide concentrations from 1.6 (red) to 9 (dark violet) μM Aβ42 in the absence or in the presence of 0.9 μM Bri2 BRICHOS. The fitting parameters are summarized in **Supplementary Table 2**.

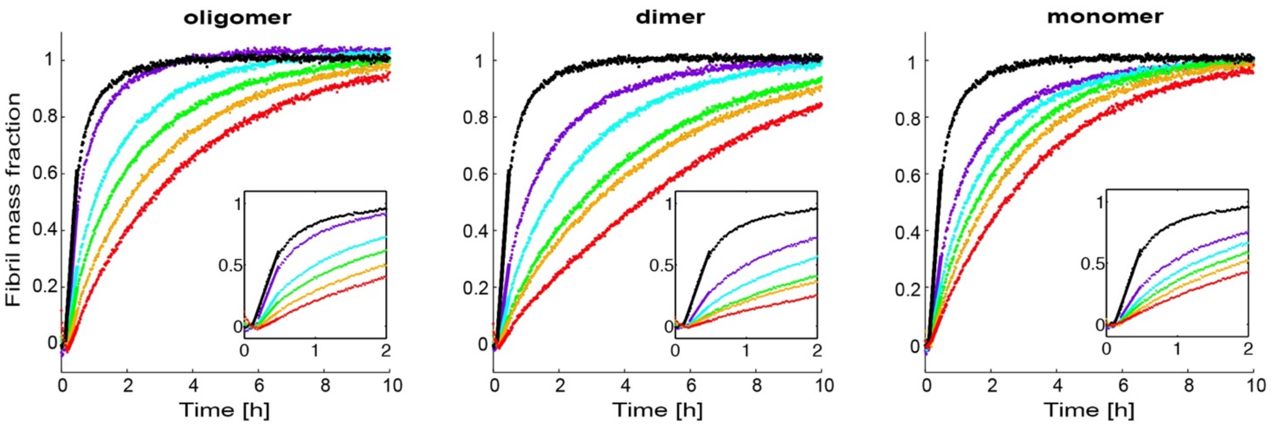

Supplementary Figure 6. Seeded aggregation traces of Aβ42 in the presence and absence of rh Bri2 BRICHOS species. Seeded aggregation traces of 3 µM Aβ42 with 0.6 µM preformed Aβ42 fibrils in the presence of 0 (black), 10 (violet), 30 (cyan), 50 (green), 70 (yellow) and 100 % (red) Bri2 BRICHOS (molar percentage referred to monomeric subunits). The inserted graphs show a zoom for the first two hours where the initial slope is determined by a linear fit to the first 30 min.

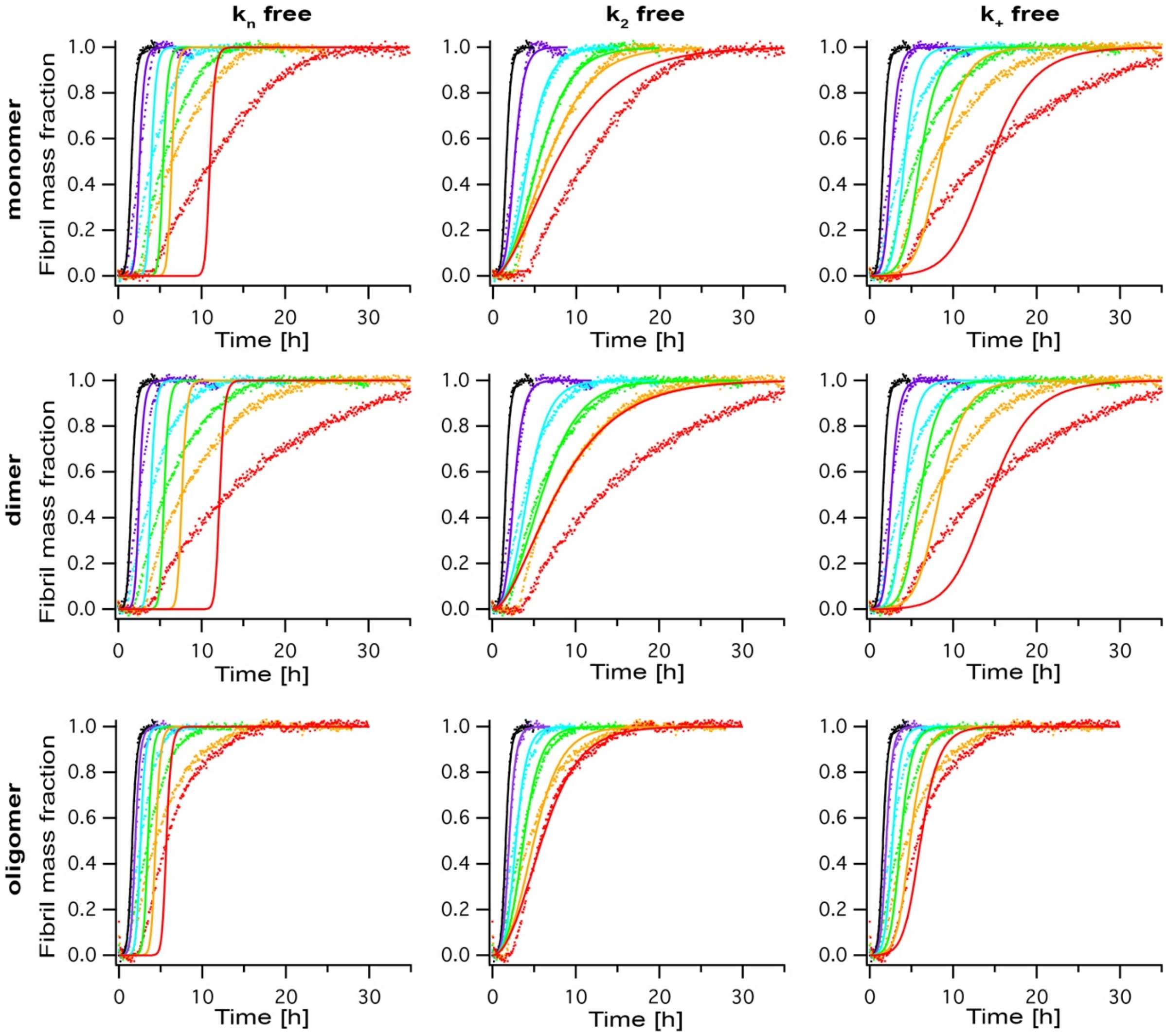

Supplementary Figure 7. Aggregation kinetics of Aβ42 in the presence of different Bri2 BRICHOS oligomer, dimer and monomer concentrations. Aggregation kinetics of 3 μM Aβ42 in the presence of different Bri2 BRICHOS concentrations (molar percentage referred to monomeric subunits): 0 (black), 10 (violet), 30 (cyan), 50 (green), 70 (yellow) or 100 % (red). The global fits (solid lines) of the aggregation traces (dots) were constrained such that only one single rate constants is the sole free fitting parameter, indicated at the top of each column. χ^2 values describing the quality of the fits are listed in **Supplementary Table 3**.

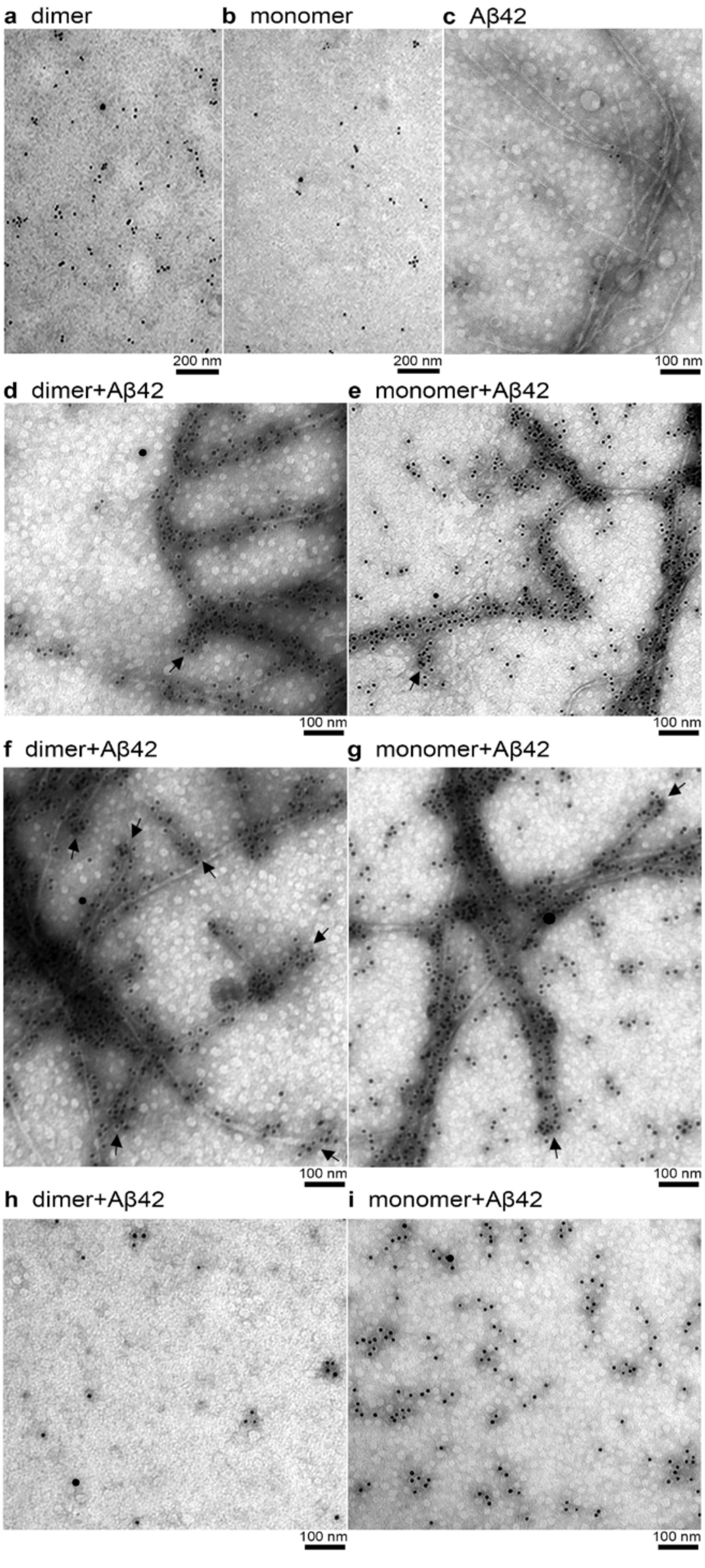
a dimer
b monomer
c Aβ42
200 nm
200 nm
100 nm
d dimer+Aβ42
e monomer+Aβ42
100 nm
100 nm
f dimer+Aβ42
g monomer+Aβ42
100 nm
100 nm
h dimer+Aβ42
i monomer+Aβ42
100 nm
100 nm

Supplementary Figure 8. Immuogold TEM of Aβ42 fibrils with and without Bri2 BRICHOS. 5 µM Aβ42 was incubated with and without 50% molar ratio of rh Bri2 BRICHOS monomer or dimer overnight at 37°C, or the same concentrations of Bri2 BRICHOS were incubated alone. The samples were treated with a Bri2 BRICHOS antibody and a gold-labeled secondary antibody and characterized by TEM. The sizes of the scale bars are indicated underneath each panel. **(a)** 50% Bri2 BRICHOS dimer alone; **(b)** 50% Bri2 BRICHOS monomer alone; **(c)** Aβ42 alone; **(d, f** and h**)** Aβ42+50% Bri2 BRICHOS dimer; **(e**, **g and i)** Aβ42+50% Bri2 BRICHOS monomer. Black arrows in **d-g** identify Aβ42 fibril ends covered by Bri2 BRICHOS.

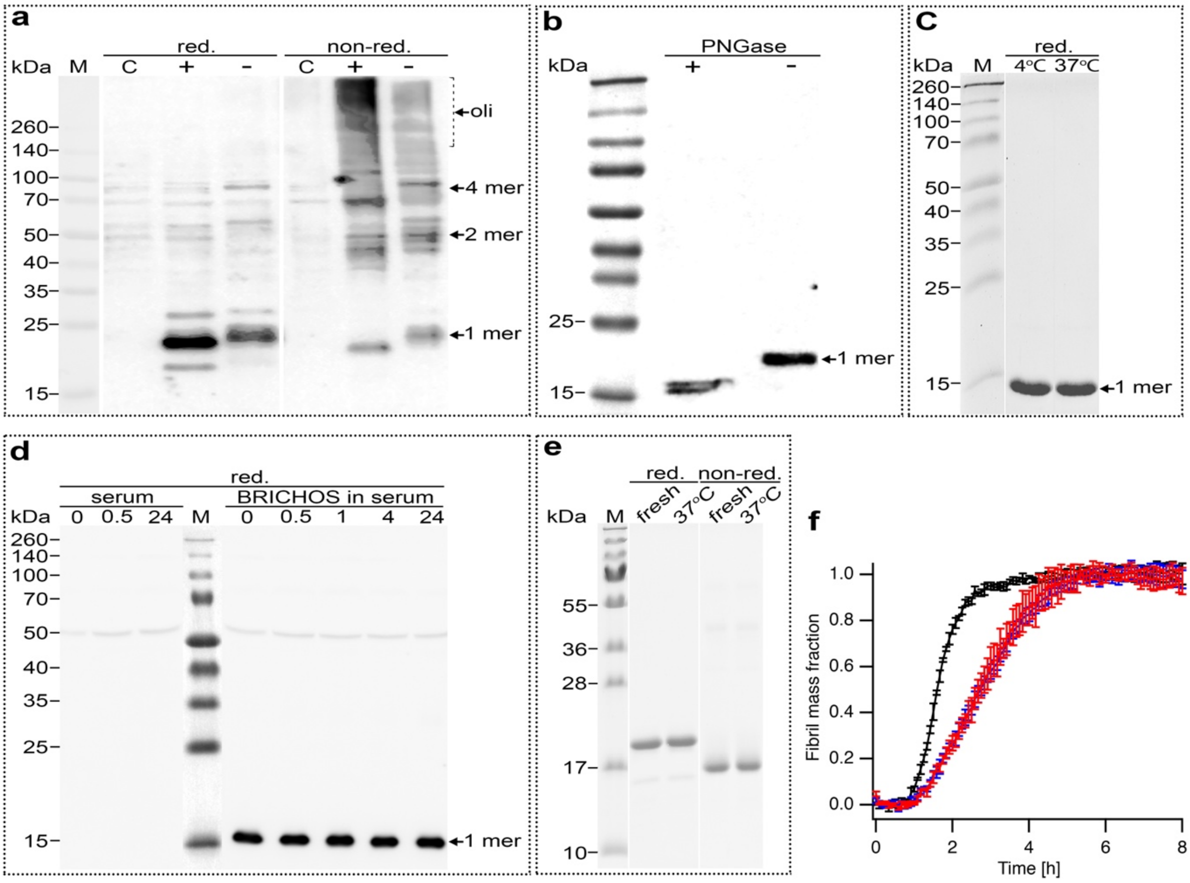

Supplementary Figure 9. Analyses of rh Bri2 BRICHOS expressed in mammalian cells, and rh Bri2 BRICHOS incubation in buffer and serum. (a) Lysates of HEK293 cells expressing rh Bri2 BRICHOS were incubated with (+) and without (-) *N*-Ethylmaleimide (NEM) overnight at 37°C, and the samples were then analyzed by Western blotting. Lane C shows analyses of non-transfected cells. **(b)** Lysates of HEK293 cells expressing rh Bri2 BRICHOS were incubated with (+) and without (-) 5 U PNGase F overnight at 37°C, and then the samples were analyzed by SDS-PAGE under reducing conditions and western blotting. **(c)** Monomeric rh Bri2 BRICHOS incubated at 4°C or 37°C overnight and then analyzed by SDS-PAGE under reducing conditions. **(d)** Monomeric rh Bri2 BRICHOS incubated in mouse serum at 37°C, or serum only, analyzed by western blotting under reducing conditions at different time points (in hours) as indicated above each lane. **(e)** Recombinant proSP-C BRICHOS was kept at -20°C (fresh) or incubated at 37°C overnight and then analyzed by SDS-PAGE under reducing and non-reducing conditions. **(f)** Fibril formation of 3 μM Aβ42 alone (black), or in the presence of 50% molar ratio of proSP-C BRICHOS kept at -20°C (red) or incubated at 37°C overnight (blue).

Supplementary Tables

Supplementary Table 1. Free thiols/molecule of Bri2 BRICHOS monomer under different conditions.

Non-reduced	Denatured	Reduced
-0.01 ± 0.06	0.03 ± 0.02	2.00 ± 0.04

Data represents mean values ± sd. of three measurements

Supplementary Table 2. Fitting parameters from global fit in **Supplementary Figure 5**.

Fitting parameter	Aβ42 alone	Monomer	Dimer	Oligomer
$\sqrt{k_n k_+}$ $[M^{-1}s^{-1}]$	7.9 ± 0.3	7.9 §	7.9 §	7.9 §
$\sqrt{k_+ k_2}$ $[M^{-3/2}s^{-1}]$	$1.32 \cdot 10^5$ *	$4.15 \cdot 10^4$	$3.03 \cdot 10^4$	$6.41 \cdot 10^4$
Relative $\sqrt{k_+ k_2}$	100 %	31 %	23 %	48 %
Relative $k_+ k_2$	100 %	10 %	5 %	24 %

* Fitting error $< 2\times10^1$, § Fixed value to Aβ42 alone

Supplementary Table 3. Assessment of quality of fits shown in **Supplementary Figure 7**.

χ^2	k_n free	k_2 free	k_+ free
**Monomer**	33.3	7.4	7.2
**Dimer**	52.5	18.2	16.6
**Oligomer**	18.0	1.6	6.1

Supplementary Table 4. Gamma oscillations power in mouse hippocampal slice preparations under different conditions shown in **Figure 5**.

Experiment	Mean ± sem [10^{-9} V2]	Number
**KA control gamma**	4.96 ± 0.41	24
**KA gamma after 50 nM Aβ42**	1.28 ± 0.17	17
**KA gamma after Aβ42 and 10 nM Bri2 BRICHOS oligomers**	1.65 ± 0.47	7
**KA gamma after Aβ42 and 50 nM Bri2 BRICHOS oligomers**	2.78 ± 0.13	7
**KA gamma after Aβ42 and 100 nM Bri2 BRICHOS oligomers**	2.47 ± 0.42	7
**KA gamma after Aβ42 and 10 nM Bri2 BRICHOS dimers**	0.98 ± 0.17	7
**KA gamma after Aβ42 and 50 nM Bri2 BRICHOS dimers**	3.45 ± 0.44	9
**KA gamma after Aβ42 and 100 nM Bri2 BRICHOS dimers**	2.94 ± 0.53	7
**KA gamma after Aβ42 and 10 nM Bri2 BRICHOS monomers**	1.21 ± 0.53	5
**KA gamma after Aβ42 and 20 nM Bri2 BRICHOS monomers**	2.69 ± 0.44	5
**KA gamma after Aβ42 and 50 nM Bri2 BRICHOS monomers**	5.68 ± 0.09	8
**KA gamma after 50 nM Bri2 BRICHOS oligomers**	5.69 ± 0.04	3
**KA gamma after 50 nM Bri2 BRICHOS dimers**	5.92 ± 0.1	3
**KA gamma after 50 nM Bri2 BRICHOS monomers**	5.81 ± 0.05	3

Supplementary Table 5. Primer sequences for NT*-Bri2 BRICHOS cloning

Primers	Sequence (from 5′to 3′)
**Sense**	TATTGAATTCCCTGGTGCCACGCGGTTCTCAGACAATTGAAGAAAATATT
**Antisense**	GGATCGGGTACCAAGCTTACAGTTTGTAAGTTTCCTTGT
